# Radial scars/complex sclerosing lesions of the breast: radiologic and clinicopathologic correlation

**DOI:** 10.1186/s12880-018-0279-z

**Published:** 2018-11-03

**Authors:** Su Min Ha, Joo Hee Cha, Hee Jung Shin, Eun Young Chae, Woo Jung Choi, Hak Hee Kim, Ha-Yeon Oh

**Affiliations:** 1 Department of Radiology, Research Institute of Radiology, Chung-Ang University Hospital, Chung-Ang University College of Medicine, 84 Heukseok-Ro, Dongjak-Gu, Seoul, 06973 Republic of Korea; 2 Department of Radiology, Research Institute of Radiology, University of Ulsan College of Medicine, Asan Medical Center, 88 Olympic-Ro 43 Gil, Songpa-Gu, Seoul, 05505 Republic of Korea; 3 Department of Radiology, Kangwon National University Hospital, 200-722 Baengnyeong-Ro 156, Chuncheon-Si, Republic of Korea

**Keywords:** Radial scar, Complex sclerosing lesion, Mammography, Ultrasound, Surgical management

## Abstract

**Background:**

We investigated the radiologic and clinical findings of radial scar and complex sclerosing lesions, and evaluated the rate of pathologic upgrade and predicting factors.

**Methods:**

From review of our institution’s database from January 2006 to December 2012, we enrolled 82 radial scars/complex sclerosing lesions in 80 women; 51 by ultrasound guided core needle biopsy, 1 by mammography-guided stereotactic biopsy, and 38 by surgical excision. The initial biopsy pathology revealed that 53 lesions were without high risk lesions and 29 were with high risk lesions. Radiologic, clinical and pathological results were analyzed statistically and upgrade rates were calculated.

**Results:**

Of the 82 lesions, 64 (78.0%) were surgically excised. After surgical excision, two were upgraded to DCIS and two were upgraded to lesions with high risk lesions. The rate of radial scar with high risk lesions was significantly higher in the surgical excision group (11.1% vs. 42.2%, *p* = 0.015), which also demonstrated larger lesion size (10.7 ± 6.5 vs. 7.1 ± 2.6 mm, *p* = 0.001). The diagnoses with high risk lesions on final pathological results showed older age (52.9 ± 6.0 years vs. 48.4 ± 6.7 years, *p* = 0.018).

**Conclusions:**

Radial scars with and without high risk lesions showed no statistically significant differences in imaging, and gave relatively low cancer upgrade rates.

**Electronic supplementary material:**

The online version of this article (10.1186/s12880-018-0279-z) contains supplementary material, which is available to authorized users.

## Background

Radial scar is characterized by stellate configuration of a fibroelastic core with entrapped ducts and lobules, and is also referred to as complex sclerosing lesion (CSL) [1]. Radial scar/CSL is diagnosed at image-guided biopsy with an incidence ranging from 0.6 to 3.7% [2, 3]. Despite being uncommon, radial scars remain important in patient management because their radiologic appearance overlaps that of invasive carcinoma and their diagnosis is challenging for radiologists [4], with the potential to be misinterpreted by pathologists as low grade invasive ductal or tubular carcinoma [5]. Radial scars can be indistinguishable from invasive carcinoma on radiologic appearance alone, often presenting as a spiculated mass or architectural distortion [6, 7]. Once diagnosis has been made with core biopsy, management is controversial because of the intrinsic malignant potential of radial scars and their coexistence with breast cancer and other high risk lesions. Radial scar is one of the proliferative categories that can coexist alongside other proliferative high risk lesions, including atypia, with each contributing to the overall upgrade rate to malignancy at excision [8–10]. As both radiology and pathology are imperfect for predicting associated malignancy, the prudence of surgical excision versus conservative management remains debatable.

Radial scar/CSL is associated with atypical proliferative lesions and has been suggested as early stage development of invasive carcinoma. The radiologically detected radial scar associated malignancy rate ranged from 10.0 to 41.0% on excision [11]. However, recent studies with carefully performed correlations between radiological and pathology findings suggest that upgrade to carcinoma on core biopsy occurs in less than 2.0% [12–15]. Furthermore, most of the lesions upgraded from radial scar are ductal carcinoma in situ (DCIS) or low grade ductal or tubular type [12, 13, 15]. The short-term follow-up of radial scars that were not excised has shown no upgrades [13, 15–18]. Thus, some insist that a subset of patients with radial scars may safely forego excision, especially in the absence of coexisting high risk lesions or other indications for excision [14, 18]. Miller et al. [15] reported an upgrade rate to invasive carcinoma of less than 1% at surgical excision of radial scar, with or without associated high risk lesions, and also revealed that the radiologic appearances of a mass or architectural distortion on mammography or ultrasound (US) are more likely to be upgraded than calcifications. Brenner et al. [16] conducted a study on one of the largest cohorts and reported an upgrade rate of 5% from a median 38 months of follow-up or by surgery, and suggested that excision of radial scar can be avoided when there is no high risk lesion on core biopsy, more than 12 biopsy specimens have been collected, and the histologic and mammographic findings are concordant. With regard to MRI of radial scar, MRI has a 97.6–100.0% negative predictive value for differentiating between benign and malignant radial scar lesions [19–23]. With the emergence of digital breast tomosynthesis, increased screening examinations, and advanced MRI, many biopsies are now performed on lesions in their earlier stages and with a smaller size, and an increasing number of mammographically occult radial scars have been detected by US, with 15.7% to 39.0% of radial scars without atypia being diagnosed [2, 24, 25]. The objective of this study was to evaluate patients with radial scar/CSL with or without high risk lesions according to management strategy such as surgical excision or follow-up of at least 5 years, comparing associated malignancy, and to identify clinical, radiologic (mammography, US, and MRI), and pathologic features predictive of upgrade.

## Methods

### Study population

Our institutional review board approved this retrospective study, and the requirement for informed consent was waived. The database of our institution was retrospectively reviewed for image-guided biopsy and breast surgery occurring between January 2006 to December 2012 to identify patients with a diagnosis of radial scar/CSL with or without high risk lesions (i.e., atypical epithelial hyperplasia, lobular neoplasia, papilloma, or atypical papilloma). Radial scar was defined as a lesion of 1.0 cm or smaller, while CSL was defined as a lesion larger than 1.0 cm. We excluded patients who were initially diagnosed with ipsilateral breast cancer or those who were followed-up for a period of less than 5 years without having undergone surgery. Finally, we enrolled 82 radial scars/CSLs in 80 women; 51 were diagnosed by US-guided core needle biopsy, one by mammography-guided stereotactic biopsy, and 38 by surgical excision.

At initial diagnosis, 53 of the lesions were considered to be without high risk lesions and 29 lesions were considered as being accompanied by high risk lesions. Of the 53 radial scars/CSLs without high risk lesions, 37 lesions (69.8%) were surgically excised and 16 lesions (30.2%) were not excised but underwent imaging follow-up of at least 5 years. Of the 29 radial scars/CSLs with high risk lesions (15 atypical epithelial hyperplasia, 7 atypically papilloma, 7 papilloma), 27 lesions (93.1%) were surgically excised and two lesions (6.9%) were not excised but underwent imaging follow-up.

We recorded whether lesions were detected during screening or diagnostic examination, and whether patients were asymptomatic or symptomatic.

An upgrade was defined as occurring when the surgical pathology was changed from 1) a lesion without high risk to a high risk lesion, DCIS, or invasive carcinoma, and 2) from lesions with high risk to DCIS or invasive carcinoma.

### Imaging technique and biopsy methods

Mammography was performed using a full-field digital mammogram unit (Senographe DS or Senographe Essential scanner, both from Generic Electric Medical System, Milwaukee, WI, USA). Two standard imaging planes were used, the mediolateral oblique and craniocaudal views.

Whole-breast US was performed using an IU22 unit (Philips Medical System, Bothell, WA, USA) equipped with a 50 mm linear array transducer with a bandwidth of 5–12 MHz. At our institution, the scanning technique for bilateral whole-breast US is standardized as follows: scanning is performed in a transverse and sagittal orientation, with the inner breast in a supine position and the outer breast in a supine oblique position with the patient’s arm raised above her head. The pectoralis muscle has to be seen on all images to ensure that the entire breast is examined. Each lesion is documented with an image of its longest dimension and an orthogonal measurement.

Dynamic contrast-enhanced MRI was performed on a 1.5 T scanner (Magnetom Avanto, Siemens Medical Solutions, Erlangen, Germany) using a dedicated 18-channel phased-array breast coil. The standard breast MRI protocol included the following pulse sequences: 1) an axial two-dimensional T2-weighted short tau inversion recovery (STIR) turbo spin-echo pulse sequence (repetition time/echo time/time interval (TR/TE/TI), 6700/74/150 ms; field of view (FOV) 300 × 300 mm; matrix size, 448 × 448; slice thickness, 5 mm); 2) pre and post-contrast-enhanced fat-saturated axial three-dimensional T1-weighted fast low angle shot volume interpolated breath-hold examination (FLASH VIBE) pulse sequences (TR/TE, 5.2/2.4 ms; FOV 340 × 340 mm; matrix size, 384 × 384; slice thickness, 0.9 mm). The six dynamic sequences (one unenhanced and five contrast-enhanced acquisitions with a temporal resolution of 59 s) were acquired before and after injection of contrast medium.

US-guided core needle biopsy was performed with a 14-gauge dual action semi-automatic core biopsy needle (Stericut with coaxial guide, TSK Laboratory, Tochigi, Japan), and a minimum of five core samples were obtained. Biopsies of mammographic findings such as asymmetry, architectural distortions, and calcifications without a corresponding US finding were performed with a stereotactic technique involving 11-gauge vacuum probes (Mammotome; Ethicon Endo-Surgery, Cincinnati, OH) on an upright stereotactic digital unit, using a Senographe Essential stereotaxic machine (General Electric Medical Systems, Milwaukee, WI).

All surgical excision was performed after US or mammography-guided wire localization, with specimen mammography being performed in cases of mammography-guided wire localization.

### Analysis of imaging findings

Mammography, US, and MRI were retrospectively reviewed (without regard to the initial clinical reporting) by two radiologists with 6 and 17 years of clinical experience in breast imaging, respectively. Each radiologist was blind to the readings of the other radiologist. When a discrepancy occurred, the two radiologists reviewed the case together and reached a consensus. Imaging features were described and assessed according to the American College of Radiology (ACR) BI-RADS 5th edition [26].

On mammography, lesions were classified into mass, calcification, mass with calcification, architectural distortion, architectural distortion with calcification, and asymmetry. For a mass, the shape was described as oval, round, or irregular, and the margin as circumscribed, obscured, microlobulated, indistinct, or spiculated. For calcifications, the distribution was analyzed as diffuse, regional, grouped, linear, or segmental, and the morphology as amorphous, coarse heterogeneous, fine pleomorphic, or punctate.

On US, the shape of each mass was described as oval, round, or irregular; the orientation as parallel or non-parallel; the margin as circumscribed, indistinct, angular, microlobulated, or spiculated; and the echo pattern as anechoic, hyperechoic, complex cystic and solid, hypoechoic, isoechoic, or heterogeneous. Posterior features were classified as no posterior feature, posterior enhancement, posterior shadowing, or a combination. The visibility of any calcification on US was also assessed.

On breast MRI for mass lesions, the margins were described as circumscribed, irregular, or spiculated, and the shape as oval, round, or irregular. The internal enhancement of each mass was classified as homogeneous, heterogeneous, rim, or dark internal septation enhancement. For non-mass lesions, the distributions were described as focal, linear, segmental, regional, multiple regions, or diffuse, while the pattern of enhancement was classified as homogeneous, heterogeneous, clumped, or clustered ring. The enhancement kinetic curve was described as Type 1 (persistent enhancement), Type 2 (plateau), or Type 3 (peak early enhancement followed by delayed washout).

### Statistical analysis

The characteristics of the patients and findings of the lesions, including upgrade rates, are summarized using number and percentage. Clinical characteristics and imaging findings were compared between lesions that underwent surgical excision (*n* = 64) and lesions that underwent follow-up (*n* = 18), with Student’s *t*-tests being used for continuous variables and *χ*^2^ tests for categorical variables. Lesions were also grouped according to the final pathological diagnosis (performed by surgical excision or follow-up of at least 5 years) as radial scar without high risk lesions (*n* = 49) or radial scar with high risk lesions (*n* = 33), and were compared using Student’s *t*-test or a Mann-Whitney test for continuous variables, and Fisher’s exact test for categorical variables. Calculations for statistical analyses were performed using SPSS software (version 23.0, IBM Corp., Armonk, NY, USA). A *P*-value of less than 0.05 was considered to indicate a statistically significant difference between groups.

## Results

**Fig. 1:**
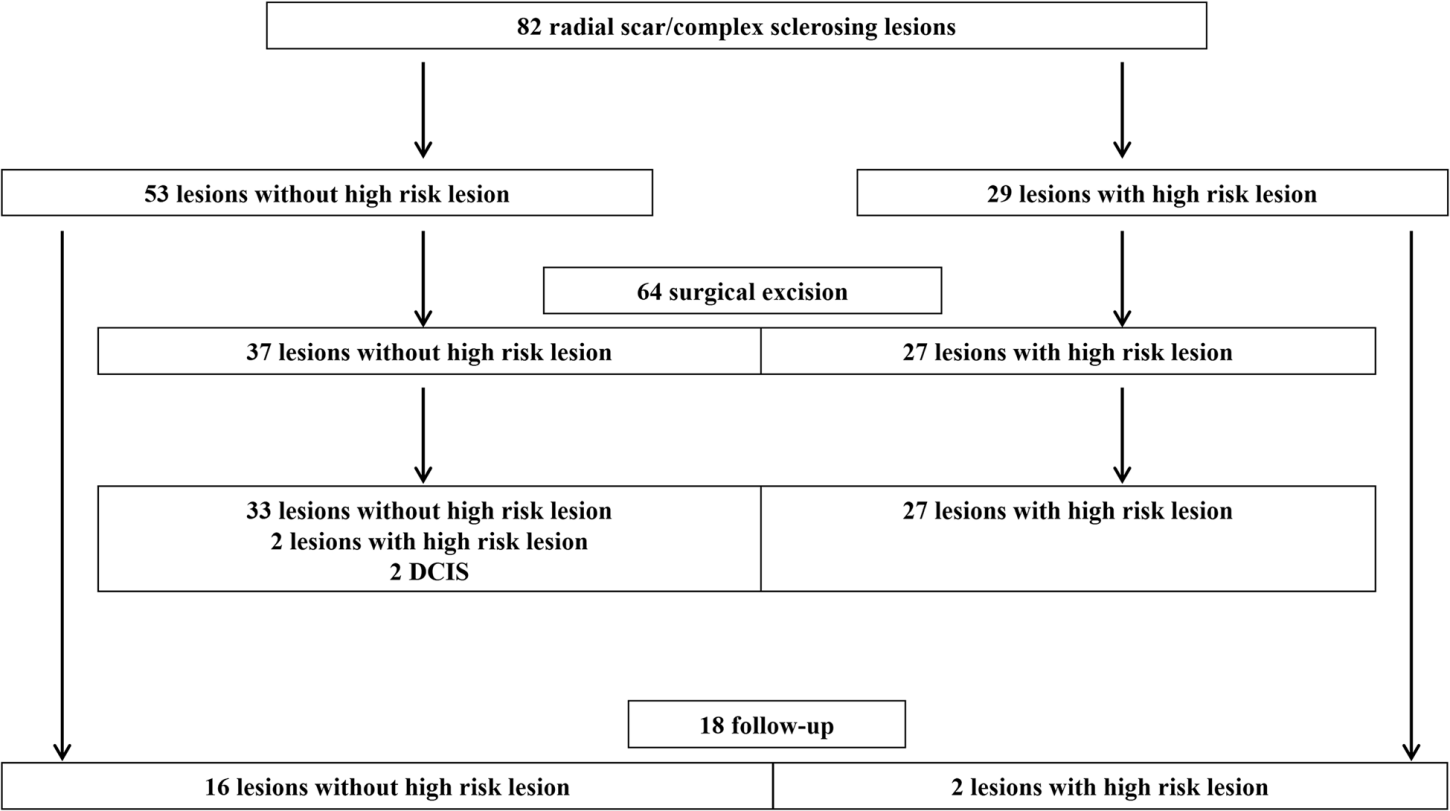
Outcomes of Radial Scar/Complex Sclerosing Lesions Diagnosed by 5-Year Follow-up and Surgery

Of the 82 lesions, 64 (78.0%) were surgically excised and 18 (22.0%) were not excised (Fig. 1). During the 5-year follow-up of the non-excised 18 lesions, there was no imaging or clinical change suggestive of an upgrade. However, two DCIS lesions (3.1%) were identified among 64 lesions that were surgically excised. In comparison with the follow-up group, the surgical excision group had a significantly higher percentage of radial scars/CSLs with high risk lesions (11.1% vs. 42.2%, *p* = 0.015) and contained lesions with a larger mean size (7.1 ± 2.6 mm vs. 10.7 ± 6.5 mm, *p* = 0.001). There were more patients with a contralateral breast malignancy such as invasive ductal cancer (*n* = 7, 70.0%), DCIS (*n* = 1, 10.0%), or tubular carcinoma (*n* = 2, 20.0%) in the operated-on group, but not statistically significant (*p* = 0.576) and were mostly screen detected without symptoms in both groups (100% and 92.2%, *p* = 0.581).

Out of all the 82 lesions in 80 patients, mammography was performed on 73 (89.0%), US on 82 (100.0%), and MRI on 13 (15.9%). Analysis of the imaging findings from mammography, US, and MRI, including the BI-RADS category, revealed no statistically significant differences between the surgical excision and follow-up groups. In both groups, masses on mammography (eleven mass only and six mass with calcification) had a mostly irregular shape (14/17, 82.4%), spiculated margin (10/17, 58.8%), and were hyperdense (10/17, 58.8%), while calcifications (four calcification only, six mass with calcification, and three architectural distortion with calcification) had a predominantly amorphous morphology (8/13, 61.5%) with regional (7/13, 53.8%) distribution. One lesion manifested as an architectural distortion only, and one as an asymmetry on mammography. On US, 79 lesions were seen as masses. Masses had mostly irregular shape (52/79, 65.8%), indistinct margin (34/79, 43.0%), and hypoechogenicity (69/79, 87.3%), without associated posterior features (62/79, 78.5%) or calcification (76/79, 96.2%). On MRI performed on thirteen lesions, six lesions (46.2%) were not seen, six lesions (46.2%) manifested as mass, and one lesion (7.6%) as non-mass enhancement. Six masses were irregular (3/6, 50%) with irregular margin (4/6, 66.7%) homogeneous enhancement (5/6, 83.3%) and showed Type 3 kinetic curve (5/6, 83.3%), and one non-mass lesion showed regional, homogeneous enhancement with Type 3 kinetic curve.

In the comparison between radial scars/CSLs with and without high risk lesions on final pathological results (Table 1), there was a greater mean age in the group with high risk lesions (52.9 ± 6.0 years; range, 33–62 years vs. 48.4 ± 6.7 years; range, 38–63 years, *p* = 0.018). There were no differences in lesion size (9.8 ± 5.9 mm vs. 10.0 ± 6.2 mm, *p* = 0.887), symptoms (*p* = 0.643), or associated malignancy in the contralateral breast (*p* = 0.717), and the findings of mammography, US and MRI (Additional file 1: Table S1).

**Table 1:** Comparisons between Radial Scars/Complex Sclerosing Lesions with and without High Risk Lesions on Final Pathological Results

Characteristics		Radial scar/CSL without high risk (*n* = 49)	Radial scar/CSL with high risk (*n* = 33)	*P*-value
Mean age		48.4 ± 6.7	52.9 ± 6.0	0.018
Follow-up		16 (32.7)	2 (6.1)	0.004
Operation		33 (67.3)	31 (93.9)	
Symptom	–	45 (91.8)	32 (97.0)	0.643
	+	4 (8.2)	1 (3.0)	
Size (mm)		9.8 ± 5.9	10.0 ± 6.2	0.887
Contralateral breast malignancy^a^		5 (10.9)	5 (15.2)	0.717

*Data* indicate the number of lesions, *Numbers in parentheses* indicate percentages

*CSL* complex sclerosing lesion, *DCIS* ductal carcinoma in situ

^a^include *DCIS*, invasive ductal carcinoma, and tubular carcinoma

**Fig. 2:**
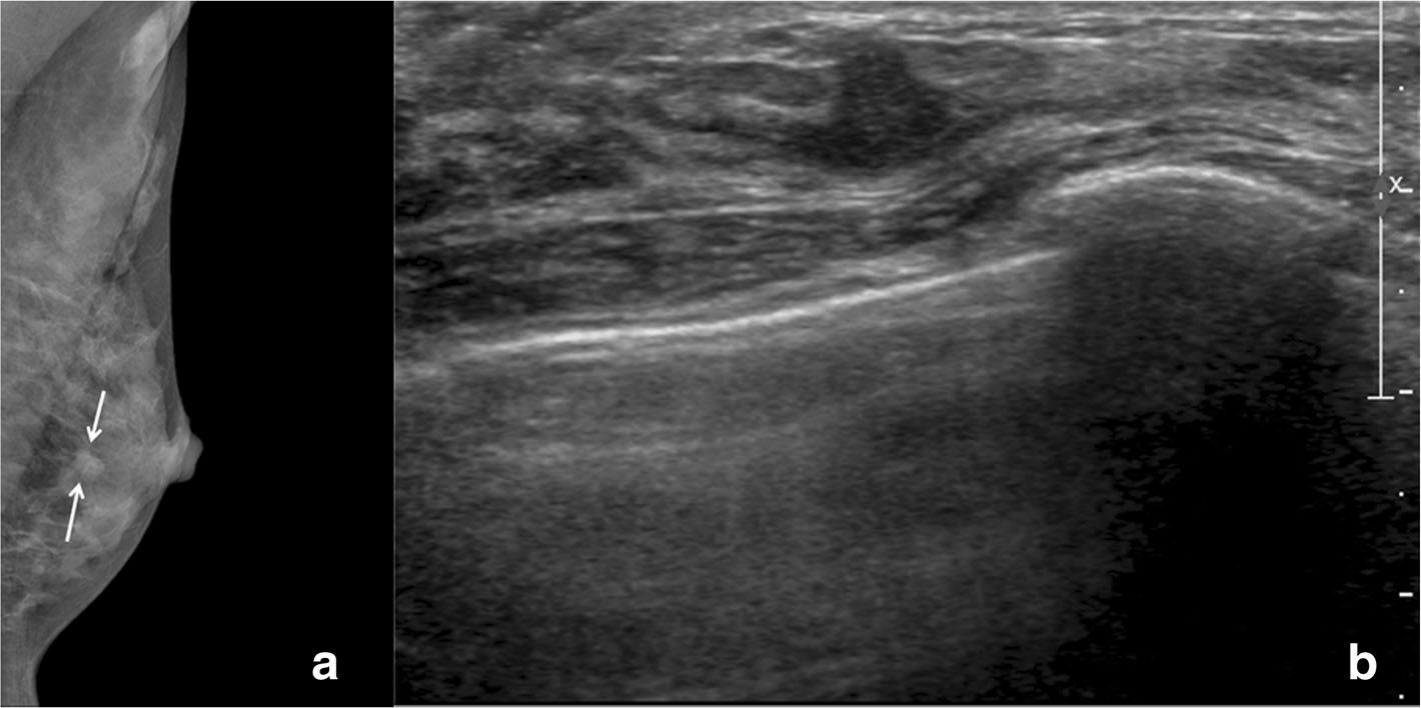
A radial scar on ultrasound guided core needle biopsy and upgraded to radial scar with atypical ductal hyperplasia after surgical excision. **a** On mammography, there is an irregular, indistinct, hyperdense mass (arrow). **b** On ultrasound, there is an irregular, non-parallel, angular, hypoechoic mass

**Fig. 3:**
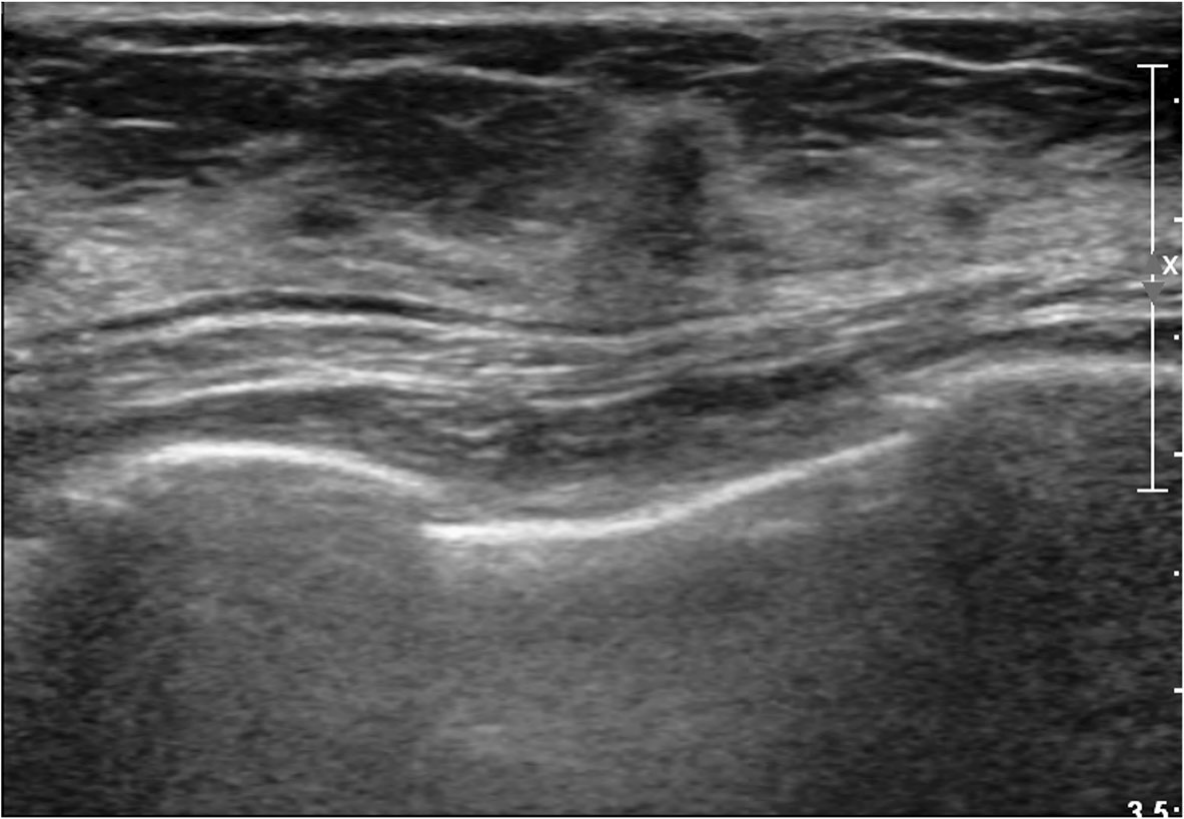
A radial scar on ultrasound- guided core needle biopsy and upgraded to ductal carcinoma in situ after surgical excision. Ultrasound shows an irregular, non-parallel, indistinct, and hypoechoic mass

Four cases (4.8%, 4/82) that were detected on the screening examination and diagnosed as radial scars/CSLs without high risk lesions on core needle biopsy were upgraded to high risk (*n* = 2) or DCIS (n = 2) after surgical excision (Table 2). The two lesions (size 1.0 cm and 1.9 cm) that were upgraded to DCIS were both occult on mammography and seen as hypoechoic masses on US, with one of these lesions appearing as non-mass enhancement with Type 3 kinetic curve on MRI (Fig. 2). The two lesions (size 5 mm and 8 mm) that were upgraded to high risk lesions (atypical ductal hyperplasia and atypical papilloma, respectively) were both hypoechoic masses on US (Fig. 3).

**Table 2:** Clinical, Radiologic, and Pathologic Data of those Radial Scars/Complex Sclerosing Lesions Upgraded after Excision

Case	Initial pathology on core biopsy	Size (mm)	Mammography	Ultrasound	MRI	Final pathology after surgical excision
1	Radial scar	5	Occult	Oval hypoechoic mass, parallel, indistinct	N/A	Radial scar with atypical ductal hyperplasia
2.	Radial scar	8	Irregular, indistinct, hyperdense mass	Irregular, non-parallel, angular, hypoechoic mass, with posterior enhancement	N/A	Radial scar with atypical papilloma
3.	Complex sclerosing lesion	19	Occult	Oval, parallel, circumscribed, hypoechoic mass	Non-mass lesion with regional, homogeneous enhancement and Type 3 kinetic curve	DCIS
4.	Radial scar	10	Occult	Irregular, non-parallel, indistinct, hypoechoic mass,	N/A	DCIS

*DCIS* ductal carcinoma in situ, *MRI* magnetic resonance imaging

## Discussion

The radial scar manifests as an architectural distortion with long thin spicules radiating from an often radiolucent central area [27]. This spiculated radiologic finding mimics malignancy and is difficult to differentiate from tubular carcinoma [28], and surgical excision has been generally recommended [29, 30]. Linda et al. agreed that the mammographic and US features of lesions diagnosed as radial scars are not predictive of the absence or presence of associated malignancy [2]. In our study, we also failed to find any radiologic features helpful for differentiating between radial scar/CSLs with and without associated high risk lesions or malignancy.

Reported upgrade rates after surgical excision have ranged from 0.0 to 40.0% because of heterogeneous study populations; a mixture of isolated radial scar with or without atypia, core and surgical excision biopsies, small sample sizes, and no comparisons between who did and did not undergo surgery [18]. Without there being any established malignancy upgrade rates or malignancy expectancy, a 2011 survey of practicing breast surgeons showed that 76.0% would recommend surgery for radial scar/CSL diagnosed on needle biopsy [31]. Similar surveys of radiologists performed at the American Roentgen Ray Society annual meetings in 2010 and 2011 showed that most radiologists would recommend surgical excision, with rates of 89.0% and 73.0%, respectively [32]. However, recent studies with careful correlations between radiology and pathology suggest that upgrade to carcinoma occurs in less than 2.0%, and advocate imaging follow-up rather than surgical excision for radial scars without high risk lesions [12–15]. Moreover, Park et al. recently reported 0.0% upgrade rate to high risk lesions or malignancy in mammographically occult radial scar/CSL diagnosed by biopsy in asymptomatic patients [33]. At the excision of both mammographically evident and occult lesions, the malignancies associated with radial scars are frequently low- or intermediate-grade DCIS, or grade 1 or 2 invasive carcinoma [2, 24, 25]. Invasive cancers in radial scars are low grade, and their biological profiles (positivity for estrogen and progesterone receptors, low proliferative index) are favorable [34]. Similarly, in our study in which 3.1% (2/64) of surgically excised lesions were upgraded to DCIS after excision, which were occult on mammography. With the low upgrade rate and favorable histopathological prognosis, conservative management with US follow-up may be recommended, rather than prompt surgical management.

Radial scar/CSL has been associated with both age and lesion size; lesions smaller than 6–7 mm or in women under the age of 40 are not correlated with cancer, but patients over 50 years of age with lesions greater than 2 cm are at a slightly higher risk [35]. Upstaging in our study was noted with radial scars/CSLs larger than 1.0 cm, and a statistically significant older age was also observed in radial scars with high risk lesions. Several risk factors have been found to be associated with upstaging of radial scar, including older age (> 50 years), postmenopausal status, larger size on radiography, and the presence of atypical hyperplasia [24]. High risk lesions such as atypical hyperplasia are generally considered to be subject to high rates of malignancy underestimation, and thereby warrant surgical excision. [36–38]. Previous studies demonstrated that radial scars with associated high risk lesions had a higher rate of upgrade to invasive or noninvasive breast cancer [16, 24]. When radial scar is found with high risk lesion, the frequency of upgrade averages 26.0%, which compares with a rate of 7.5% for those without [39]. Several previous studies have described the use of large-gauge vacuum-assisted needle, and have suggested that if there is no association with atypia and radiologic and histologic findings are concordant, further surgery is not required [16]. However, other studies have not shown significant difference in upgrade rates between vacuum-assisted biopsy devices of differing sizes [40]. Knowledge on the outcomes of radial scars that did not undergo surgery is important for making decisions on optimal management. In our study, none of the un-excised lesions that were followed for at least 5 years resulted in any subsequent malignant lesions. This is consistent with previous study in which there were no malignant lesions during a follow-up period of up to 11 years [41], although the study excluded any atypical proliferative lesions. Resetkova et al. [18] also reported no subsequent carcinoma at a median follow-up of 29 months in patients who did not have radial scar excised.

Our study has limitations. It is a retrospective study and has relatively small sample size. Further prospective study with larger population should validate this claim. Also, there was a substantial proportion of US-guided biopsy and one case of mammography-guided biopsy, any meaningful statistical analysis regarding needle size with upgrade rate could not be carried out. Lastly, due to few cases examined on mammography and MRI, this may introduce bias to the imaging finding interpretations.

## Conclusions

In conclusion, radial scar with or without high risk lesions was associated with a low upgrade rate (3.1%) to DCIS on surgery or 5 year follow-up, with no lesions being upgraded to invasive carcinoma. Therefore, excision of radial scars to diagnosis of carcinoma is not warranted. With the emergence of more sophisticated imaging modalities and an increasing number of screening examinations, the question of how to manage these lesions has arisen as a clinical dilemma. In the future, the management of patients with radial scars should be refined according to clinical data, radiologic findings, and the coexistence of high risk lesions, to minimize unnecessary interventions. Radial scar/CSL with associated high risk lesions or larger lesions may undergo surgical excision to maximize the early detection of coexistent invasive carcinoma, evaluate the presence of high risk lesions, and provide guidance on chemoprevention to reduce the future cancer risk.

## Additional file

Additional file 1: **Table S1.** Imaging features of the radial scar/complex scelrosing lesion with and without associated high risk lesions. (DOCX 20 kb)

## Abbreviations

CSL: Complex sclerosing lesion
DCIS: Ductal carcinoma in situ
MRI: Magnetic resonance imaging
US: Ultrasound

## References

[1] Eusebi V, Millis RR (2010). Epitheliosis, infiltrating epitheliosis, and radial scar. Semin Diagn Pathol.

[2] Linda A, Zuiani C, Furlan A, Londero V, Girometti R, Machin P, Bazzocchi M (2010). Radial scars without atypia diagnosed at imaging-guided needle biopsy: how often is associated malignancy found at subsequent surgical excision, and do mammography and sonography predict which lesions are malignant?. Am J Roentgenol.

[3] Sohn VY, Causey MW, Steele SR, Keylock JB, Brown TA (2010). The treatment of radial scars in the modern era--surgical excision is not required. Am Surg.

[4] Meyer JE, Christian RL, Lester SC, Frenna TH, Denison CM, DiPiro PJ, Polger M (1996). Evaluation of nonpalpable solid breast masses with stereotaxic large-needle core biopsy using a dedicated unit. Am J Roentgenol.

[5] Rabban JT, Sgroi DC (2004). Sclerosing lesions of the breast. Semin Diagn Pathol.

[6] Cohen MA, Sferlazza SJ (2000). Role of sonography in evaluation of radial scars of the breast. AJR Am J Roentgenol.

[7] Cawson JN, Nickson C, Evans J, Kavanagh AM (2010). Variation in mammographic appearance between projections of small breast cancers compared with radial scars. J Med Imaging Radiat Oncol.

[8] Braakhuis BJ, Tabor MP, Kummer JA, Leemans CR, Brakenhoff RH (2003). A genetic explanation of Slaughter's concept of field cancerization: evidence and clinical implications. Cancer Res.

[9] Chai H, Brown RE (2009). Field effect in cancer-an update. Ann Clin Lab Sci.

[10] Cohen MA, Newell MS (2017). Radial scars of the breast encountered at Core biopsy: review of histologic, imaging**,** and Management Considerations. AJR Am J Roentgenol.

[11] Fasih T, Jain M, Shrimankar J, Staunton M, Hubbard J, Griffith CD (2005). All radial scars/complex sclerosing lesions seen on breast screening mammograms should be excised. Eur J Surg Oncol.

[12] Conlon N, D'Arcy C, Kaplan JB, Bowser ZL, Cordero A, Brogi E, Corben AD (2015). Radial scar at image-guided needle biopsy: is excision necessary?. Am J Surg Pathol.

[13] Donaldson AR, Sieck L, Booth CN, Calhoun BC (2016). Radial scars diagnosed on breast core biopsy: frequency of atypia and carcinoma on excision and implications for management. Breast.

[14] Matrai C, D'Alfonso TM, Pharmer L, Drotman MB, Simmons RM, Shin SJ (2015). Advocating nonsurgical Management of Patients with Small, incidental radial scars at the time of needle Core biopsy: a study of 77 cases. Arch Pathol Lab Med.

[15] Miller CL, West JA, Bettini AC, Koerner FC, Gudewicz TM, Freer PE, Coopey SB, Gadd MA, Hughes KS, Smith BL (2014). Surgical excision of radial scars diagnosed by core biopsy may help predict future risk of breast cancer. Breast Cancer Res Treat.

[16] Brenner RJ, Jackman RJ, Parker SH, Evans WP, Philpotts L, Deutch BM, Lechner MC, Lehrer D, Sylvan P, Hunt R (2002). Percutaneous core needle biopsy of radial scars of the breast: when is excision necessary?. Am J Roentgenol.

[17] Becker L, Trop I, David J, Latour M, Ouimet-Oliva D, Gaboury L, Lalonde L (2006). Management of radial scars found at percutaneous breast biopsy. Can Assoc Radiol J.

[18] Resetkova E, Edelweiss M, Albarracin CT, Yang WT (2011). Management of radial sclerosing lesions of the breast diagnosed using percutaneous vacuum-assisted core needle biopsy: recommendations for excision based on seven years' of experience at a single institution. Breast Cancer Res Treat.

[19] Linda A, Zuiani C, Furlan A, Lorenzon M, Londero V, Girometti R, Bazzocchi M (2012). Nonsurgical management of high-risk lesions diagnosed at core needle biopsy: can malignancy be ruled out safely with breast MRI?. AJR Am J Roentgenol.

[20] Pediconi F, Occhiato R, Venditti F, Fraioli F, Napoli A, Votta V, Laghi A, Catalano C, Passariello R (2005). Radial scars of the breast: contrast-enhanced magnetic resonance mammography appearance. Breast J.

[21] Razek AA, Gaballa G, Denewer A, Nada N (2010). Invasive ductal carcinoma: correlation of apparent diffusion coefficient value with pathological prognostic factors. NMR Biomed.

[22] Razek AA, Lattif MA, Denewer A, Farouk O, Nada N (2016). Assessment of axillary lymph nodes in patients with breast cancer with diffusion-weighted MR imaging in combination with routine and dynamic contrast MR imaging. Breast cancer.

[23] Abdel Razek AA, Gaballa G, Denewer A, Tawakol I (2010). Diffusion weighted MR imaging of the breast. Acad Radiol.

[24] Andacoglu O, Kanbour-Shakir A, Teh YC, Bonaventura M, Ozbek U, Anello M, Ganott M, Kelley J, Dirican A, Soran A (2013). Rationale of excisional biopsy after the diagnosis of benign radial scar on core biopsy: a single institutional outcome analysis. Am J Clin Oncol.

[25] Nassar A, Conners AL, Celik B, Jenkins SM, Smith CY, Hieken TJ (2015). Radial scar/complex sclerosing lesions: a clinicopathologic correlation study from a single institution. Ann Diagn Pathol.

[26] American College of Radiology (2013). Breast imaging reporting and data system (BI-RADS).

[27] Bianchi S, Giannotti E, Vanzi E, Marziali M, Abdulcadir D, Boeri C, Livi L, Orzalesi L, Sanchez LJ, Susini T (2012). Radial scar without associated atypical epithelial proliferation on image-guided 14-gauge needle core biopsy: analysis of 49 cases from a single-Centre and review of the literature. Breast.

[28] Lopez-Medina A, Cintora E, Mugica B, Opere E, Vela AC, Ibanez T (2006). Radial scars diagnosed at stereotactic core-needle biopsy: surgical biopsy findings. Eur Radiol.

[29] Berg WA (2004). Image-guided breast biopsy and management of high-risk lesions. Radiol Clin N Am.

[30] Osborn G, Wilton F, Stevens G, Vaughan-Williams E, Gower-Thomas K (2011). A review of needle core biopsy diagnosed radial scars in the welsh breast screening Programme. Ann R Coll Surg Engl.

[31] Krishnamurthy S, Bevers T, Kuerer H, Yang WT (2012). Multidisciplinary considerations in the management of high-risk breast lesions. Am J Roentgenol.

[32] Georgian-Smith D, Lawton TJ (2012). Variations in physician recommendations for surgery after diagnosis of a high-risk lesion on breast core needle biopsy. Am J Roentgenol.

[33] Park VY, Kim EK, Kim MJ, Yoon JH, Moon HJ (2016). Mammographically occult asymptomatic radial scars/complex Sclerosing lesions at ultrasonography-guided Core needle biopsy: follow-up can be recommended. Ultrasound Med Biol.

[34] Mokbel K, Price RK, Mostafa A, Williams N, Wells CA, Perry N, Carpenter R (1999). Radial scar and carcinoma of the breast: microscopic findings in 32 cases. Breast.

[35] Sloane JP, Mayers MM (1993). Carcinoma and atypical hyperplasia in radial scars and complex sclerosing lesions: importance of lesion size and patient age. Histopathology.

[36] McGhan LJ, Pockaj BA, Wasif N, Giurescu ME, McCullough AE, Gray RJ (2012). Atypical ductal hyperplasia on core biopsy: an automatic trigger for excisional biopsy?. Ann Surg Oncol.

[37] Elsheikh TM, Silverman JF (2005). Follow-up surgical excision is indicated when breast core needle biopsies show atypical lobular hyperplasia or lobular carcinoma in situ: a correlative study of 33 patients with review of the literature. Am J Surg Pathol.

[38] Sohn V, Arthurs Z, Herbert G, Keylock J, Perry J, Eckert M, Fellabaum D, Smith D, Brown T (2007). Atypical ductal hyperplasia: improved accuracy with the 11-gauge vacuum-assisted versus the 14-gauge core biopsy needle. Ann Surg Oncol.

[39] Sun J, Liu X, Zhang Q, Hong Y, Song B, Teng X, Yu J (2016). Papillary thyroid carcinoma treated with radiofrequency ablation in a patient with hypertrophic cardiomyopathy: a case report. Korean J Radiol.

[40] Eby PR, Ochsner JE, DeMartini WB, Allison KH, Peacock S, Lehman CD (2009). Frequency and upgrade rates of atypical ductal hyperplasia diagnosed at stereotactic vacuum-assisted breast biopsy: 9-versus 11-gauge. Am J Roentgenol.

[41] Hou Y, Hooda S, Li Z (2016). Surgical excision outcome after radial scar without atypical proliferative lesion on breast core needle biopsy: a single institutional analysis. Ann Diagn Pathol.

