# Supplementary material for: Radial scars/complex sclerosing lesions of the breast: radiologic and clinicopathologic correlation
Source: BMC Med Imaging. 2018 Nov 3;18:39. doi: 10.1186/s12880-018-0279-z (PMC6215659; doi:10.1186/s12880-018-0279-z)
Supplement: Supplementary file 1 — Table S1. Imaging features of the radial scar/complex scelrosing lesion with and without associated high risk lesions. (DOCX 20 kb) [file 12880_2018_279_MOESM1_ESM.docx]

**Table S1** Imaging features of the radial scar/complex scelrosing lesion with and without associated high risk lesions.

| Characteristics | Radial scar/CSL without high risk (n=49) | Radial scar /CSL with high risk (n=33) | *P* value |
| --- | --- | --- | --- |
| **Mammography (n=73)** | n = 41 | n =32 |  |
| Lesion type |  |  |  |
| Occult | 25 (61.0) | 22 (68.8) | 0.537 |
| Mass only | 7 (17.1) | 4 (12.5) |  |
| Calcification only | 5 (12.2) | 2 (6.3) |  |
| Mass with calcification  Architectural distortion | 2 (4.9 )  2 (4.8) | 4 (12.5)  0 (0.0) |  |
| Mass shape |  |  | >0.999 |
| Oval | 1 (11.1) | 0 (0.0) |  |
| Round | 1 (11.1) | 1 (12.5) |  |
| Irregular | 7 (77.8) | 7 (87.5) |  |
| Mass margin |  |  | >0.999 |
| Circumscribed | 1 (11.1) | 0 (0.0) |  |
| Non-Circumscribed | 8 (88.9) | 7 (100.0) |  |
| Calcification morphology |  |  | 0.633 |
| Amorphous | 5 (71.4) | 3 (50.0) |  |
| Coarse heterogeneous | 1 (14.3) | 0 (0.0) |  |
| Fine pleomorphic | 1 (14.3) | 2 (33.3) |  |
| Benign | 0 (0.0) | 1 (16.7) |  |
| Calcification distribution |  |  | 0.286 |
| Regional | 5 (71.4) | 2 (33.3) |  |
| Grouped | 2 (28.6) | 4 (66.7) |  |
| **Ultrasound (n=82)** | n=49 | n=33 |  |
| Occult | 1 (2.0) | 2 (6.1) |  |
| Mass shape |  |  | 0.404 |
| Oval | 12 (25.0) | 10 (32.3) |  |
| Round | 2 (4.2) | 3 (9.7) |  |
| Irregular | 34 (70.8) | 18 (58.1) |  |
| Orientation |  |  | 0.889 |
| Parallel | 24 (50.0) | 15 (48.4) |  |
| Non-Parallel | 24 (50.0) | 16 (51.6) |  |
| Mass margin |  |  | 0.424 |
| Circumscribed | 3 (6.3) | 4 (12.9) |  |
| Non-Circumscribed | 45 (93.8) | 27 (87.1) |  |
| Echo pattern |  |  | 0.672 |
| Complex | 1 (2.1) | 1 (3.2) |  |
| Hypoechoic | 41 (85.4) | 28 (90.3) |  |
| Isoechoic | 3 (6.3) | 0 (0.0) |  |
| Heterogeneous | 3 (6.3) | 2 (6.5) |  |
| Posterior echo features |  |  | 0.879 |
| Absent | 39 (81.3) | 23 (74.2) |  |
| Enhancement | 3 (6.3) | 3 (9.7) |  |
| Shadowing | 5 (10.4) | 4 (12.9) |  |
| Combined | 1 (2.1) | 1 (3.2) |  |
| Calcification |  |  | 0.558 |
| Present | 1 (2.1) | 2 (6.5) |  |
| Absent | 47 (97.9) | 29 (93.5) |  |
| **MRI (n=13)** | n=5 | n=8 |  |
| Occult | 2 (40.0) | 4 (50.0) |  |
| Lesion Type |  |  | >0.999 |
| Mass | 3 (100.0) | 3 (75.0) |  |
| Non-Mass | 0 (0.0) | 1 (25.0) |  |
| Mass shape |  |  | >0.999 |
| Oval | 1(33.3) | 1 (33.3) |  |
| Round | 0 (0.0) | 1 (33.3) |  |
| Irregular | 2 (66.7) | 1 (33.3) |  |
| Mass margin  Circumscribed | 1 (33.3) | 1 (33.3) | >0.999 |
| Non-Circumscribed | 2 (66.7) | 2 (66.7) |  |
| Mass internal enhancement |  |  | >0.999 |
| Homogeneous | 2 (66.7) | 3 (100.0) |  |
| Heterogeneous | 1 (33.3) | 0 (0.0) |  |
| Enhancement curve type |  |  | 0.429 |
| Type 1: Persistent | 1 (33.3) | 0 (0.0) |  |
| Type 2: Plateau  Type 3: Washout | 0 (0.0)  2 (66.7) | 0 (0.0)  4 (100.0) |  |

Data indicate the number of lesions. Numbers in parentheses indicate percentages.

CSL: complex sclerosing lesion; MRI: magnetic resonance imaging
